# Patterns of headache in patients with antiphospholipid syndrome in relation to autoantibodies

**DOI:** 10.1186/s10194-026-02381-4

**Published:** 2026-05-11

**Authors:** Radwa Soliman, Youssra Gadallah Mahfouz, Rania Gamal Anwar El-Skaan, Warda Abdelfattah Shehata Mohamed

**Affiliations:** 1 Neurology and Psychiatry Department, Faculty of Medicine, Ain Shams University, Cairo, Egypt; 2 Internal Medicine Department, Immunology Division, Ain Shams University, Cairo, Egypt; 3 Obstetrics and Gynecology Department, Ain Shams University, Cairo, Egypt; 4 Internal Medicine Department, Rheumatology Division, Ain Shams University, Cairo, Egypt

**Keywords:** Antiphospholipid syndrome, Antiphospholipid antibodies, Primary, Migraine, Headache, Tension type headache, Triple positivity

## Abstract

**Background:**

The interplay between Antiphospholipid syndrome (APS) and migraine is very controversial; migraine is believed to be the most common neurologic symptom in APS, and there are claims that migraineurs have abnormally high Antiphospholipid antibodies (aPL), yet the reports differ widely. Nevertheless, APS could lead to various other types of headaches; both primary and secondary, leaving patients susceptible to exacerbation of symptoms, overlapping of headaches, and atypical presentations.

**Aim of the work:**

To describe types and characteristics of headache in a cohort of patients with antiphospholipid syndrome (APS) in comparison to healthy controls. And investigate their possible relation with antiphospholipid antibodies (aPL) and MRI brain findings in patients with APS.

**Methods:**

We recruited 76 patients with APS consecutively, between February 2024 and August 2025. From Ain shams university hospital; neurology, internal medicine, and obstetric and gynecology departments, along with 60 matched controls. Patients and controls underwent neurological examination and were evaluated for headache according to the International Headache Society criteria (IHS), Impact and severity were assessed by Migraine Disability Assessment (MIDAS) and Headache Impact Test (HIT). All participants had a brain MRI.

**Results:**

A total of 76 patients; 70 females and 6 males, mean age for patients at presentation was 35 ± 8.73 years; mean disease duration was 5 ± 3 years; mean Body mass index (BMI) was 22.38 ± 1.46. Headache was reported by 87% of patients; of which 16 patients suffered secondary type headaches (21%); and 50 patients had primary headaches (65.7%); with migraine being the most common (35%). Anticardiolipin (ACL) antibodies were the most common aPL detected (76.4%) in APS patients. MRI brain was normal in 86% of APS patients with primary headaches, Meanwhile, APS patients with triple positive antibodies showed significantly more abnormal MRI findings (*p* = 0.04). Headache severity was significantly more in APS patients (*p* = 0.04) Factors affecting headache severity were APS disease duration, BMI, triple aPL positivity (*p = *0.03, 0.00, 0.02 respectively).

**Conclusion:**

Primary and secondary types of headaches are frequently reported in Patients with APS, primary headache being more common; migraine in particular. APS patients had significantly more severe headaches. MRI brain was normal in the majority of APS patients with primary headaches and healthy controls. Yet, patients with triple positive antibodies showed more abnormal MRI findings. APS disease duration, BMI, triple aPL were independent factors for headache severity.

**Supplementary Information:**

The online version contains supplementary material available at 10.1186/s10194-026-02381-4.

## Background

Antiphospholipid syndrome (APS) is an autoimmune disease characterized by antibodies against the antiphospholipid complex, which presents as thrombotic and/or obstetric manifestations. APS may occur as a primary condition or secondary to other autoimmune diseases, most commonly systemic lupus erythematosus (SLE) [1].

The nervous system is one of the main targets of APS, and various neurologic manifestations have been linked with the disease [2] Headache and especially migraine was frequently reported in patients with APS and is one of the clinical features associated with antiphospholipid antibodies (aPL) as described early by Hughes. However, this remains controversial, with many studies questioning the association [3].

Furthermore, headache was reported as an initial presentation of catastrophic antiphospholipid syndrome (CAPS), which is a rare but potentially fatal manifestation; where rapid vascular occlusion occurs simultaneously in over three locations, thus necessitating early identification and management [4, 5]. 

Although various studies stated that headaches, namely migraine, are the most frequently reported symptoms in APS, the prevalence varied markedly across them [6–8]. Meanwhile, some studies reported an abnormal presence of aPL in otherwise normal patients with migraine, suggesting an involvement of these autoantibodies in migraine crisis [9]. Which could strengthen the claims that immunological dysfunction has considerable links with migraine pathogenesis [10–12].

Whilst there are reports of APS from Africa, their main focus was obstetric and thrombotic manifestations. Hence there is a real need to evaluate the wide range of its presentation and assess its impact as it remains far under diagnosed [13].

Thus, study was conducted in order to describe types and characteristics of headaches in a cohort of patients with APS in comparison to healthy controls. And to investigate the possible relation between aPL and headache features.

### Patients and methods

This is a hospital-based case control study conducted in Ain Shams University Hospital, a tertiary hospital in Cairo. Adult APS patients from both genders were recruited consecutively from Neurology, Internal medicine, and obstetric and gynecology clinics, between February 2024 and August 2025; APS was diagnosed according to 2006 Revised Sapporo Criteria [14]. Patients with primary and secondary APS were included. Along with 60 age and sex matched healthy controls, who were recruited from visitors of inpatients in the surgery hospital. Controls were interviewed initially to exclude any neurological, autoimmune diseases, or vascular incidents and risk factors. They were tested for aPL to ensure negativity.

All included participants underwent full neurological history and examination and a semi structured interview for headache according to the international headache society [15] headache severity was assessed by Arabic versions of Migraine Disability Assessment (MIDAS) [16] and Headache Impact Test (HIT) [17], Depression was assessed by Arabic version of Beck’s Depression Inventory (BDI) [18]. We excluded APS patients with grave presentations, as well as APS patients secondary to SLE during high or very high disease activity diagnosed by rheumatologist via SLE disease activity index (SLEDAI- 2 K) [19]. To ensure we could truly rely on their self-reporting for headache history and to avoid headache related to disease activity.

Brain MRI, MR angiography (MRA), and MR venography (MRV) were performed on a General Electric optima 1.5 T clinical scanner (General Electric Medical Systems, MR450W) according to standard imaging protocol.

Initially, MRI findings were categorized as normal or abnormal; where lesions found were, white matter hyper intensities (WMH) defined as high signal intensity on T2-weighted images and FLAIR images, Ischemia defined as increased signal of diffusion weighted imaging (DWI) and decreased apparent diffusion coefficient (ADC) values in acute stage, high signal intensity on T2 and FLAIR weighted images in subacute stage and low signal intensity on T1 weighted images with high signal intensity on T2-weighted images in chronic stage. Dural venous sinus thrombosis defined as filling defect in affected venous sinus on MRV [20]. 

Then patients with abnormal MRI who only had few focal WMH that aren’t associated with mass effect within the deep, subcortical, periventricular, or infratentorial areas were individually categorized [21]. 

Antiphospholipid antibodies (aPL) tested were lupus anticoagulant (LA), anti-cardiolipin (ACL) IgG/IgM, anti-b2-glycoprotein-I (anti-β2-GPI) IgG/IgM. Tests were performed with Enzyme-linked Immunosorbent assay (ELISA) technique, according to the current recommendations. Values for the 3 aPL were considered positive if determined at least twice and at 12 weeks apart time interval [22]. 

### Statistical methods

Analysis of data was done using SPSS program version 27. Quantitative data were presented using minimum, maximum, mean and standard deviation. Qualitative data were presented using count and percentage. Student t test was used to compare quantitative data between two independent groups. One way ANOVA test was used to compare quantitative data between more than two independent groups. Chi square and Fisher exact tests were used to compare qualitative data between different groups. *P* value less than or equal to 0.05 was considered statistically significant.

## Results

### Demographic data

70 females and 6 males; mean age for the patients at presentation was 35 ± 8.73 years; mean disease duration was 5 ± 3 years; 74% of patients had secondary APS, where most of them were secondary to SLE (Table 1).

**Table 1:** Clinical data and headache types in patients with APS

Clinical data				Min.	Max.		Mean		SD
Age (years)				22.00	55.00		35.04		8.73
Disease duration (years)				0.40	12.00		5.09		2.93
BMI				20.00	24.00		22.38		1.39
				**N**			**%**		
Sex	Male			6			7.9%		
	Female			70			92.1%		
Smoking	No			59			77.6%		
	Yes			17			22.4%		
Type of APS	Primary APS			20			26.3%		
	Secondary APS			56			73.7%		
Type of APS	Thrombotic			64			84.2%		
	Obstetric			12			15.8%		
Cause of Secondary APS	SLE			41			73.2%		
	RA			10			17.9%		
	MCTD			4			7.1%		
	Sjogren			1			1.8%		
				N			%		
MRI brain	Normal			57			75.0%		
	Non-specific WMH			9			11.8%		
	Abnormal MRI			10			13.2%		
		MRI brain						X2*	P value
		Normal		Non-specific WMH		Abnormal MRI			
		N	%	N	%	N	%		
Type of headache	Migraine	18	90.0%	2	10.0%	0	0.0%	33.38 FE	**< 0.001**
	Migraine with aura	4	57.1%	3	42.9%	0	0.0%		
	TTH	20	90.9%	2	9.1%	0	0.0%		
	Secondary headache	4	25.0%	2	12.5%	10	62.5%		

SLE: Systemic lupus erythematosus, RA: Rheumatoid arthritis, MCTD: Mixed connective tissue disease, TTH: Tension type headache, TAC: Trigeminal autonomic cephalgia, CVT: Cerebral venous thrombosis, WMH: White matter hyperintensities

### Secondary headaches

**Fig. 1:**
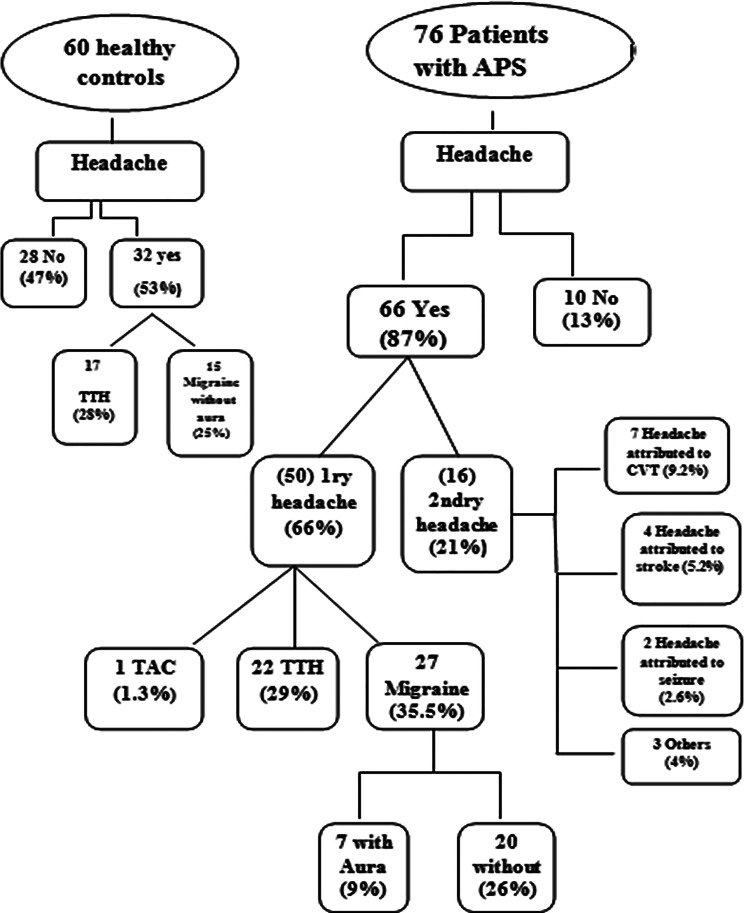
Shows stratification of APS patients according to their headache status and type, along with types of headaches reported by controls. TTH: Tension type headache. TAC: Trigeminal autonomic cephalgia

Out of 76 patients 66 reported headaches (87%); of which 16 patients were found to have secondary headaches (21%); where cerebral venous thrombosis was the most common cause (Fig. 1).

### Primary headaches

After excluding patients with secondary headaches, 60 APS patients were compared to 60 age and gender matched controls; in terms of types and characteristics of primary headaches, as well as depression and MRI brain (Table 2).

**Table 2:** Comparison between APS patients with 1ry headaches and controls:

		APS cases		Controls		t*	*P* value
		Mean	SD	Mean	SD		
Age (years)		33.92	8.03	35.18	9.33	0.75	0.45
BMI		22.38	1.46	22.55	1.28	0.65	0.52
		N	%	N	%	X^2**^	P value
Sex	Male	5	10.0%	5	8.3%	0.09 FE	1.00
	Female	45	90.0%	55	91.7%		
Smoking	No	40	80.0%	48	80.0%	0.00	1.00
	Yes	10	20.0%	12	20.0%		
		**APS cases (60)**		**Controls (60)**		X^2**^	P value
With primary headache		N	%	N	%		
		50	83.3%	32	53.3%		
Type of headache	Migraine	20	40.8%	15	46.9%	5.19 FE	0.08
	Migraine with aura	7	14.3%	0	0.0%		
	TTH	22	44.9%	17	53.1%		
		APS cases with 1ry headache		Controls		t*	P value
		Mean	SD	Mean	SD		
BDI		25.94	18.00	17.55	15.75	2.61	**0.01**
MIDAS		12.14	7.58	8.07	6.61	2.97	**0.004**
HIT 6		55.26	9.15	44.88	12.03	5.13	**< 0.001**
		N	%	N	%	X^2**^	P value
BDI	Minimal	15	30.0%	32	53.3%	6.78	**0.01**
	Mild	7	14.0%	8	13.3%		
	Moderate	6	12.0%	5	8.3%		
	Severe	22	44.0%	15	25.0%		
MIDAS	No disability	15	30.0%	33	55.0%	9.23	**0.003**
	Mild	8	16.0%	10	16.7%		
	Moderate	12	24.0%	10	16.7%		
	Severe	15	30.0%	7	11.7%		
HIT6	Little or no impact	14	28.0%	38	63.3%	14.92	**< 0.001**
	Some impact	11	22.0%	10	16.7%		
	Substantial impact	11	22.0%	5	8.3%		
	Severe impact	14	28.0%	7	11.7%		
		N	%	N	%		
MRI brain	Normal	43	86.0%	57	95.0%	2.67 FE	0.18
	Non-specific WM changes	7	14.0%	3	5.0%		

*Student t test **Chi square test

APS patients who suffered primary headaches represented (65.7%) of cohort; with migraine being the most commonly reported type (35.5%), Tension type headache (TTH) (28.9%), and Trigeminal autonomic cephalgia (TAC) (1.3%) (Fig. 1).

As regards primary headaches, APS patients reported more frequent migraines and migraine with aura than matched controls who reported more TTH, yet statistically insignificant. Moreover, patients diagnosed with APS had significantly more depression and severe headaches via both MIDAS and HIT (*p = *0.04,* p < *0.001, respectively) Table 2.

### Brain imaging

Both APS patients with primary headaches and controls showed no statistically significant difference as regards their MRI brain findings; patients had 86% normal MRI and controls had 95%. Table 2.

Yet, WMH increased significantly across APS patients with increased headache impact via HIT (*p = *0.04) Table 3 and has been further proven by multivariate analysis (*p = *0.04) Table 7 Moreover, comparing MRI brain in migraine APS patients with and without aura showed us that WMH are more common in APS patients who have migraine with aura (43%) than migraine without aura (10%). Predictably, MRI across APS patients with 2ndry types of headaches showed statistically significant abnormal findings (*p = *0.001) Table 1.

**Table 3:** Relation between impact & severity of headache and MRI findings, and aPL:

		HIT6 score									
		Little or no impact		Some impact		Substantial impact		Severe impact			
		*N*	%	*N*	%	*N*	%	*N*	%	X^2**^	*P* value
MRI brain	Normal	7	100.0%	4	80.0%	4	80.0%	1	33.3%	8.89 FE	**0.04**
	Non-specific WMH	0	0.0%	1	20.0%	0	0.0%	2	66.7%		
	Abnormal MRI	0	0.0%	0	0.0%	1	20.0%	0	0.0%		
		**HIT6**								X^2*^	P value
		Little or no impact		Some impact		Substantial impact		Severe impact			
		N	%	N	%	N	%	N	%		
ACL IgM	Positive	21	80.8%	11	78.6%	14	93.3%	11	52.4%	8.22 FE	0.08
	Negative	5	19.2%	3	21.4%	1	6.7%	10	47.6%		
ACL IgG	Positive	17	68.0%	12	85.7%	11	84.6%	15	75.0%	1.95 FE	0.58
	Negative	8	32.0%	2	14.3%	2	15.4%	5	25.0%		
LA	Positive	16	61.5%	11	78.6%	6	42.9%	12	57.1%	3.82	0.44
	Negative	10	38.5%	3	21.4%	8	57.1%	9	42.9%		
b2 Glycoprotein I IgM	Positive	10	43.5%	7	53.8%	8	57.1%	13	65.0%	2.06	0.16
	Negative	13	56.5%	6	46.2%	6	42.9%	7	35.0%		
b2 Glycoprotein I Ig G	Positive	9	39.1%	7	53.8%	8	57.1%	13	65.0%	3.03	0.09
	Negative	14	60.9%	6	46.2%	6	42.9%	7	35.0%		
		**MIDAS score for severity**									
		No disability		Mild		Moderate		Severe			
		N	%	N	%	N	%	N	%	X^2**^	P value
MRI brain	Normal	8	100.0%	1	100.0%	3	75.0%	4	57.1%	9.35 FE	0.08
	Non-specific WM changes	0	0.0%	0	0.0%	0	0.0%	3	42.9%		
	Abnormal MRI	0	0.0%	0	0.0%	1	25.0%	0	0.0%		
		**MIDAS score for severity**									
		No disability		**Mild**		**Moderate**		**Severe**			
		**N**	**%**	**N**	**%**	**N**	**%**	**N**	**%**	**X** ^2**^	**P value**
ACL IgM	Negative	3	37.5%	0	0.0%	2	50.0%	1	14.3%	2.36 FE	0.69
	Positive	5	62.5%	1	100.0%	2	50.0%	6	85.7%		
ACL IgG	Negative	0	0.0%	0	0.0%	0	0.0%	1	14.3%	2.94 FE	1.00
	Positive	6	100.0%	1	100.0%	3	100.0%	6	85.7%		
LA	Negative	0	0.0%	0	0.0%	0	0.0%	5	71.4%	10.30 FE	**0.002**
	Positive	8	100.0%	1	100.0%	4	100.0%	2	28.6%		
b2 Glycoprotein I IgM	Negative	1	20.0%	0	0.0%	0	0.0%	5	71.4%	4.96 FE	0.15
	Positive	4	80.0%	0	0.0%	3	100.0%	2	28.6%		
b2 Glycoprotein I Ig G	Negative	1	20.0%	0	0.0%	0	0.0%	5	71.4%	4.96 FE	0.15
	Positive	4	80.0%	0	0.0%	3	100.0%	2	28.6%		

**Chi square test (FE: Fisher exact)

### Medications

Amongst treatment categories received by APS patients were antiplatelets (88%), anticoagulation (46%), glucocorticoids (21%); with a number of patients receiving more than one category. Yet we couldn’t find a correlation between any of the treatment categories received and headache severity. Table 4.

**Table 4:** Medication categories in APS patients and their relation to headache severity (MIDAS):

Medication		*N*				%					
AP	Yes	67				88.2%					
	No	9				11.8%					
AC	Yes	35				46.1%					
	No	41				53.9%					
HCQ	Yes	40				52.6%					
	No	36				47.3%					
GC	Yes	16				21.1%					
	No	60				78.9%					
MTX	Yes	8				10.5%					
	No	68				89.5%					
		**MIDAS**								X^2*^	P value
		**No disability**		**Mild**		**Moderate**		**Severe**			
		N	%	N	%	N	%	N	%		
AP	Yes	21	75.0%	12	92.3%	17	94.4%	17	100.0%	6.46 FE	0.06
	No	7	25.0%	1	7.7%	1	5.6%	0	0.0%		
AC	Yes	14	50.0%	8	61.5%	9	50.0%	4	23.5%	5.02	0.17
	No	14	50.0%	5	38.5%	9	50.0%	13	76.5%		
HCQ	Yes	11	39.2%	8	61.5%	12	66.6%	9	52.9%	4.81	0.19
	No	17	60.8%	5	38.5%	6	33.4%	8	47.1%		
GC	Yes	6	21.4%	5	38.5%	2	11.1%	3	17.6%	3.32 FE	0.35
	No	22	78.6%	8	61.5%	16	88.9%	14	82.4%		
MTX	Yes	6	21.4%	1	7.7%	0	0.0%	1	5.9%	5.13 FE	0.11
	No	22	78.6%	12	92.3%	18	100.0%	16	94.1%		

*Chi square test (FE: Fisher exact)

AP: Antiplatelet, AC: Anticoagulation, HCQ: Hydroxychloroquine, GC: Glucocorticoids, MTX: Methotrexate

### APS type and aPL

Comparing 1ry and 2ndry APS there was a significant difference across types of 1ry headaches; migraine being more common in 1ry APS. (*p = *0.045) Yet there was no difference in headache severity or MRI findings. While comparing thrombotic and obstetric APS; we found no significant difference as regard headache types or severity Table 5.

**Table 5:** Comparison between APS types; primary vs. secondary & thrombotic vs. obstetric cases:

		Primary APS		Secondary APS		t*	*P* value
		Mean	SD	Mean	SD		
BMI		21.90	1.37	22.55	1.36	1.84	0.07
		N	%	N	%	X^2**^	P value
Headache	No	4	20.0%	6	10.7%	1.11 FE	0.44
	Yes	16	80.0%	50	89.3%		
Type of headache	Primary	15	93.8%	35	70.0%	3.72 FE	0.09
	Secondary	1	6.3%	15	30.0%		
Type of primary headache	Migraine	11	68.8%	16	32%	7.26 FE	**0.045**
	TTH	4	25.0%	18	36.0%		
	TAC	0	0.0%	1	2.0%		
BDI	Minimal	7	35.0%	22	39.3%	1.65 FE	0.68
	Mild	4	20.0%	8	14.3%		
	Moderate	1	5.0%	8	14.3%		
	Severe	8	40.0%	18	32.1%		
MIDAS	No disability	8	40.0%	20	35.7%	4.35 FE	0.22
	Mild	1	5.0%	12	21.4%		
	Moderate	4	20.0%	14	25.0%		
	Severe	7	35.0%	10	17.9%		
HIT6	Little or no impact	7	35.0%	19	33.9%	2.76 FE	0.43
	Some impact	5	25.0%	9	16.1%		
	Substantial impact	5	25.0%	10	17.9%		
	Severe impact	3	15.0%	18	32.1%		
MRI brain	Normal	16	80.0%	41	73.2%	1.61 FE	0.47
	Non-specific WMH	3	15.0%	6	10.7%		
	Abnormal MRI	1	5.0%	9	16.1%		
Number of positive antibodies	Single	5	25.0%	18	32.1%	0.37	0.83
	Double	8	40.0%	21	37.5%		
	Triple	7	35.0%	17	30.4%		
		**Type of APS**				t*	P value
		**Obstetric (12)**		**Thrombotic (64)**			
		Mean	SD	Mean	SD		
BDI		31.67	20.24	19.70	17.10	2.16	0.03
MIDAS		10.83	8.00	10.88	7.61	0.02	0.99
HIT 6		51.50	11.67	53.95	11.49	0.68	0.50
		N	%	N	%	X^2**^	P value
Headache	No	3	25.0%	7	10.9%	1.75 FE	0.19
	Yes	9	75.0%	57	89.1%		
Type of headache	Migraine	6	66.7%	14	24.6%	7.35 FE	0.10
	Migraine with aura	1	11.1%	6	10.5%		
	TTH	2	22.2%	20	35.1%		
	TN	0	0.0%	1	1.8%		
Type of headache	Primary	9	100.0%	41	71.9%	3.34 FE	0.10
	Secondary	0	0.0%	16	28.1%		
BDI	Minimal	2	16.7%	27	42.2%	3.44 FE	0.31
	Mild	2	16.7%	10	15.6%		
	Moderate	2	16.7%	7	10.9%		
	Severe	6	50.0%	20	31.3%		
MIDAS	No disability	5	41.7%	23	35.9%	0.81 FE	0.92
	Mild	1	8.3%	12	18.8%		
	Moderate	3	25.0%	15	23.4%		
	Severe	3	25.0%	14	21.9%		
HIT6	Little or no impact	4	33.3%	22	34.4%	0.48 FE	0.95
	Some impact	2	16.7%	12	18.8%		
	Substantial impact	3	25.0%	12	18.8%		
	Severe impact	3	25.0%	18	28.1%		
Number of positive antibodies	Single	3	25.0%	20	31.3%	0.81 FE	0.73
	Double	6	50.0%	23	35.9%		
	Triple	3	25.0%	21	32.8%		

*Student t test **Chi square test (FE: Fisher Exact)

As regards possible correlation of aPL in APS patients and their headache, Anticardiolipin (ACL) antibodies were the most common aPL detected (76.4%) (Supplementary data 1). LA antibody differed significantly between 1ry and 2ndry headaches (*p = *0.003) Table 5.

And although its status showed significant difference across headache severity measured by MIDAS; being less in patients with severe headache (*p = *0.002) Table 3. It didn’t show significance by multivariate analysis (*p = *0.8) Table 7 or by comparing the mean MIDAS score between LA-positive and LA-negative patients (Supplementary data 2).

As for number of positive aPL there was a significant difference as regards MRI findings across patients with triple positive antibodies; they showed more abnormal findings. There was also a significant difference between number of positive antibodies and types of headaches, whether it be primary vs. secondary headache, or type of primary headache (*p = *0.04, *p* = 0.048, respectively). Table 6.

**Table 6:** Primary and secondary headaches in relation to aPL and correlates of number of positive aPL with headache:

		Type of headache						X^2**^	P value
		Primary			Secondary				
		N	%		N	%			
ACL IgM	Positive	36	72.0%		13	81.3%		0.54 FE	0.53
	Negative	14	28.0%		3	18.8%			
ACL IgG	Positive	37	78.7%		12	75.0%		0.10 FE	0.74
	Negative	10	21.3%		4	25.0%			
LA	Positive	25	51.0%		13	81.3%		4.54	**0.03**
	Negative	24	49.0%		3	18.8%			
b2 Glycoprotein I IgM	Positive	25	55.6%		9	56.3%		0.002	0.96
	Negative	20	44.4%		7	43.8%			
b2 Glycoprotein I Ig G	Positive	24	53.3%		9	56.3%		0.04	0.84
	Negative	21	46.7%		7	43.8%			
		**Number of positive antibodies**						**X** ^2**^	**P value**
		**Single**		**Double**		**Triple**			
		N	%	N	%	N	%		
Type of APS	Primary APS	5	21.7%	8	27.6%	7	29.2%	0.37	0.83
	Secondary APS	18	78.3%	21	72.4%	17	70.8%		
BDI	Minimal	11	47.8%	11	37.9%	7	29.2%	7.47 FE	0.28
	Mild	4	17.4%	4	13.8%	4	16.7%		
	Moderate	2	8.7%	1	3.4%	6	25.0%		
	Severe	6	26.1%	13	44.8%	7	29.2%		
MIDAS	No disability	9	39.1%	8	27.6%	11	45.8%	5.76 FE	0.46
	Mild	4	17.4%	4	13.8%	5	20.8%		
	Moderate	4	17.4%	8	27.6%	6	25.0%		
	Severe	6	26.1%	9	31.0%	2	8.3%		
HIT6	Little or no impact	9	39.1%	10	34.5%	7	29.2%	11.33	0.07
	Some impact	1	4.3%	9	31.0%	4	16.7%		
	Substantial impact	4	17.4%	7	24.1%	4	16.7%		
	Severe impact	9	39.1%	3	10.3%	9	37.5%		
MRI brain	Normal	18	78.3%	22	75.9%	17	70.8%	11.31	**0.01**
	Non-specific WMH	3	13.0%	6	20.7%	0	0.0%		
	Abnormal MRI	2	8.7%	1	3.4%	7	29.2%		
Type of headache	Primary	16	80.0%	23	88.5%	11	55.0%	6.67	**0.04**
	Secondary	4	20.0%	3	11.5%	9	45.0%		
Type of primary headache	Migraine	8	40%	15	57.7%	4	20%	11.27	**0.048**
	TTH	8	40.0%	8	30.8%	6	30.0%		
	TAC	0	0.0%	0	0.0%	1	5.0%		

*One Way ANOVA test **Chi square test (FE: Fisher exact)

**Table 7:** Multivariate analysis for factors associated with severity and impact of headache:

**MIDAS score**	**Unstandardized Coefficients**		**Standardized Coefficients**	**Significance**	**95.0% Confidence Interval for B**	
	**B**	**Std. Error**	**Beta**		**Lower Bound**	**Upper Bound**
Disease duration (years)	-0.657	0.302	-0.291	**0.033**	-1.259	-0.055
BMI	0.692	0.087	1.173	**0.000**	0.519	0.866
Abnormal MRI brain	0.833	2.625	0.023	0.752	-4.399	6.065
Triple positivity of Abs	-4.399	1.940	-0.187	**0.026**	-8.266	-0.0532
LA positivity	0.419	2.227	0.025	0.851	-4.022	4.860
**HIT score**	**Unstandardized Coefficients**		**Standardized Coefficients**	**Significance**	**95.0% Confidence Interval for B**	
	**B**	**Std. Error**	**Beta**		**Lower Bound**	**Upper Bound**
Disease duration (years)	-1.219	0.482	-0.131	**0.014**	-2.180	-0.259
BMI	2.601	0.139	1.065	**0.000**	2.323	2.878
Abnormal MRI brain	10.552	4.189	0.070	**0.014**	2.201	18.904
Triple positivity of Abs	− 0.346	3.096	-0.004	0.911	-6.518	5.827
LA positivity	0.357	3.569	0.005	0.921	-6.760	7.474

Moreover, multivariate analysis showed that triple positivity as well as APS disease duration, and BMI are independent factors for headache severity. (*p = *0.02, 0.03, 0.00 respectively) Table 7.

## Discussion

Over the past few decades, many studies investigated the possible interplay between primary headaches and APS. Since, different types of primary headaches, especially migraine and TTH, are amongst the most frequently reported manifestations [23].

Which doesn’t only apply for APS but also to other autoimmune CNS disorder [24] and systemic autoimmune diseases including SLE, rheumatoid arthritis (RA) and others [25], such diseases are notorious for leading to a wide range of neurological manifestations including different types of headache.

### Primary headache

In the current study, 50 APS patients and 32 controls reported primary headache; migraine was the most common type in APS (35%) followed by TTH (29%), While for controls TTH was the most common type (28%).

Such increased prevalence of primary headaches in APS patients, especially migraine, is in accordance with previous studies that showed Migraine has growing evidence of autoimmune-mediated pathogenesis and is the most commonly reported type of headache in APS/aPL-positive patients [26, 27]. Presenting in over 35% of adult APS population [28] with studies reporting the association of aPLs positivity in migraine patients [29, 30]. and others suggesting a greater risk to develop ischemic stroke for women with migraine, APS being a culprit in the pathological process [31].

Few Egyptian studies investigated primary headache in systemic autoimmune diseases and concluded migraine and TTH are common in patients with SLE, yet their data conflicted in terms of the relation between primary headaches and disease activity [32, 33].

In our cohort, one patient suffered TAC who had APS secondary to mixed connective tissue disease (MCTD); which points to previous reports that Trigeminal neuralgia (TN) is the most common neurological manifestation of MCTD [34]. And can also be the presenting symptom in MCTD and various CTD [35]. This is crucial, since TN and TAC overlap significantly which has frequently led to misdiagnosis [36] Thus, patients with TN, TAC or other types of primary headache, who show no improvement on best medical management, should be properly examined for underlying systemic autoimmune diseases.

### Secondary headaches

Were reported in 21% of included APS patients; with cerebral venous thrombosis (CVT) being the most common cause. Moreover 30% of patients with secondary headaches had secondary APS; most commonly secondary to SLE, which is tightly linked to a wide range of neurological manifestations and thus to different types of both secondary and primary headaches [33]. This adds to the complexity of headache evaluation in this group; where patients can have overlapping causes and types of headache, let al.one the adverse events of medications.

### APS type and aPL

We found no differences between patients with 1ry and 2ndry APS besides the frequency of primary headache types amongst them, where migraine was the most frequent in 1ry APS patients. While patients with 2ndry APS had more frequent TTH and TAC, this can be understood considering that patients with 2ndy APS suffer additional symptoms related to their systemic autoimmune disease and can be directly related to their headache type.

Anticardiolipin (ACL) antibodies were the most common aPL detected in our APS patients, but we couldn’t find any correlation between it and migraine, which goes with previous studies that stated its weak or nonexistent association with migraine [37–39].

Meanwhile, LA antibody differed significantly between 1ry and 2ndry headaches. Yet it showed no significant correlation with headache severity, which could be in line with previous studies that reported LA was not significantly associated with migraine patients [40, 41]. 

Number of positive aPL showed significant difference in types of headache, whether it is primary or secondary, or type of primary headache. Moreover, it correlated significantly with MRI findings, since APS patients with triple positive antibodies showed more abnormal MRI findings.

It’s worth noting that, although the number of positive Abs in APS is considered a crucial prognostic factor; where triple positivity act as an independent and strong factor of thrombotic and obstetric relapses and complications, and is even linked to CAPS [4, 42], and [43]. It hasn’t been investigated enough in APS patients with primary headaches.

### Body mass index

We found BMI to be an independent factor for headache impact and severity; which is in line with a number of studies that have linked obesity to migraine, and suggested that higher BMI could increase migraine severity [44, 45],

### Imaging

We found brain MRI to be essentially normal in the majority of APS patients with primary headaches as well as controls, with only 14% of APS patients and 5% of controls showing few nonspecific WMH, which agrees with other studies that investigated MRI findings in migraine patients [46, 47], APS patients with migraine [26], and SLE patients with migraine [33].

Yet comparing migraine APS patients with and without aura showed us that WMH are more common in migraine with aura which is in accordance with other studies suggesting WMHs are more frequently observed in patients with long standing disease duration, aura, and increased headache frequency. [48–49]

### Medications

We couldn’t find a correlation between any of the treatment categories received by patients and headache severity, including antiplatelets and anticoagulation; which is of value since migraine has been previously linked to hypercoagulability [50], thus some studies suggest a potential benefit for these medications [51], while others claim that the evidence is still inadequate, and that effectiveness depends on a range of factors; most importantly proper clinical phenotyping [52].

Finally, APS patients, in particular those secondary to systemic autoimmune diseases, can suffer a wide range of headaches. Since the overlap between various pathogenic mechanisms could exacerbate headache or alter its characteristics [53] leading to atypical presentations. This wouldn’t only increase patients’ suffering and impact their overall quality of life but may also delay diagnosis and obscure proper management. Besides the added economic burden it imposes, since multiple comorbidities, chronic use of medications were amongst the key factors associated with increasing the cost of headache management [54, 55]. thus more studies are needed to develop tools, whether clinical, radiological, or laboratorial that can facilitate phenotyping and managing those patients.

## Limitations

One major limitation in our study would be attributed to bias; both recruitment and recall. Since APS can have very serious presentations, leaving patients with more grave manifestations unable to participate. Also, the cross-sectional study type could hinder our ability to generalize our findings. Moreover, we were challenged by APS patients who reported different or overlapping types of headaches simultaneously. Finally, we couldn’t screen all patients for fibromyalgia, although it can be common in patients with chronic autoimmune disease and may cause or exacerbate their headache.

### Future research and implications

It could be beneficial to consider screening for underling autoimmune diseases, especially aPL, in selected migraine phenotypes namely migraine with aura, or persistent migraine who show no improvement on best medical management. Moreover, future research is needed with long term studies; that can follow up these patients and assess the impact of APS, other comorbidities, as well as their medications on their headache symptoms and response to management.

## Conclusion

Primary headaches and migraine in particular are common in APS. Yet, secondary headaches are also frequent and should always be considered at first for fear of their life-threatening complications. Severity and impact of primary headaches are significantly higher in APS patients than controls. Though, conventional brain MRI studies are usually normal. Triple positivity of aPL significantly differs across headache types and is more linked to MRI brain abnormalities.

## Electronic Supplementary Material

Below is the link to the electronic supplementary material.

Supplementary Material 1

## Data Availability

The datasets generated during and/or analyzed during the current study are available from the corresponding author on reasonable request.

## Abbreviations

APS: Antiphospholipid syndrome
aPL: antiphospholipid antibodies
ACL: Anti-cardiolipin
Anti-β2-GPI: Anti-b2-glycoprotein-I
BMI: Body Mass Index
CVT: Cerebral venous thrombosis
MCTD: Mixed connective tissue disease
LA: Lupus anticoagulant
RA: Rheumatoid arthritis
SLE: Systemic lupus erythematosus
TTH: Tension type headache
TAC: Trigeminal Autonomic Cephalgia
TN: Trigeminal Neuralgia
