# Supplementary material for: Patterns of headache in patients with antiphospholipid syndrome in relation to autoantibodies
Source: J Headache Pain. 2026 May 11;27(1):144. doi: 10.1186/s10194-026-02381-4 (PMC13188423; doi:10.1186/s10194-026-02381-4)
Supplement: Supplementary file 1 — Supplementary Material 1 [file 10194_2026_2381_MOESM1_ESM.docx]

***Supplementary data***

**(Supplementary data 1):**

**Antiphospholipid auto antibodies (aPL) across APS patients:**

|  | | N | % |
| --- | --- | --- | --- |
| ACL IgM | Positive | 57 | 75.0% |
|  | Negative | 19 | 25.0% |
| ACL IgG | Positive | 55 | 76.4% |
|  | Negative | 17 | 23.6% |
| LA | Positive | 45 | 60.0% |
|  | Negative | 30 | 40.0% |
| b2 Glycoprotein I IgM | Positive | 38 | 54.3% |
|  | Negative | 32 | 45.7% |
| b2 Glycoprotein I Ig G | Positive | 37 | 52.9% |
|  | Negative | 33 | 47.1% |

**(Supplementary data 2)**

**Comparing MIDAS according to Lupus anticoagulant antibody status:**

|  | Lupus Anticoagulant | | | | t* | P value |
| --- | --- | --- | --- | --- | --- | --- |
|  | Positive | | Negative | |  |  |
|  | Mean | SD | Mean | SD |  |  |
| MIDAS | 9.62 | 7.48 | 12.47 | 7.59 | 1.60 | 0.11 |

*Student t test
